# How local IRBs view central IRBs in the US

**DOI:** 10.1186/1472-6939-12-13

**Published:** 2011-06-23

**Authors:** Robert Klitzman

**Affiliations:** 1 Department of Psychiatry, Columbia University, 1051 Riverside Drive #15, New York, NY, USA

## Abstract

**Background:**

Centralization of IRB reviews have been increasing in the US and elsewhere, but many questions about it remain. In the US, a few centralized IRBs (CIRBs) have been established, but how they do and could operate remain unclear.

**Methods:**

I contacted 60 IRBs (every fourth one in the list of the top 240 institutions by NIH funding), and interviewed leaders from 34 (response rate = 55%) and an additional 12 members and administrators.

**Results:**

These interviewees had often interacted with CIRBs, but supported local reviews, and offered advantages and disadvantages of each. Interviewees argued that local IRBs can provide "local knowledge" of subjects and PIs, and "curbside consults" with PIs, facilitating mutual trust. PIs may interact more fully and informally, and hence effectively with local IRBs. IRBs also felt additional responsibility to protect "their own" subjects. Respondents mentioned a few advantages of CIRBs (e.g., CIRBs may streamline reviews), though far more rarely and cursorily. Overall, interviewees were wary of CIRBs, which they saw as varying widely in quality, depending on who happened to be members. Both local and centralized IRBs appear to have unintended consequences. For instance, discrepancies arose between IRBs that appeared to reflect differences in institutional culture and history, and personalities of chairs and/or vocal members, more than in local community values *per se*, and thus do not seem to be the intent of the regulations. While some critics see CIRBs as solutions to many IRB problems, critical tradeoffs and uncertainties emerge.

**Conclusions:**

These data have critical implications for future policy and research. Debates need to evolve beyond simply a binary discussion of whether CIRBs should replace local IRBs, to examine how and to what degree different models might operate, and what the relative advantages and disadvantages of each are. While some critics see CIRBs as panaceas, certain problems appear likely to continue. Careful consideration needs to be given to whether the advantages of local IRBs outweigh the problems that result, and whether a system can be developed that provides these benefits, while avoiding the disadvantages of local IRBs.

## Background

Centralization of reviews by Institutional Review Boards (IRBs) in the US, and Research Ethics Committees (RECs), as they are called in many other countries, have been increasing in several areas of the world, but many questions about this trend remain. The European Union (EU) has sought to harmonize reviews of clinical trials across RECs in member countries, yet responses within individual countries have varied [1-5]. Within EU countries, central, regional, and local RECs often exist, and tensions between these have arisen, including debates on how, and to what degree to unify processes and decisions [1].

In the US as well, several centralized IRBs (CIRBs) have been established, generating controversies and questions about how they do and should operate. CIRBs can be either governmental bodies or private for-profit commercial entities. These two varieties differ in certain key regards - e.g., in purpose, and potential conflicts of interest. Yet similarities exist in that both have been established as alternatives to perceived limitations of local IRBs.

Critics have argued that the current system of review is flawed [6]. In particular, documented discrepancies in IRB reviews of multi-site studies have led many critics to call for far more use of CIRBs - presumably through governmental or other non-profit mechanisms [7]. Such discrepancies can require changes in protocols that delay research, and can make data from different sites difficult to compare.

Yet in the US, CIRBs, though proposed over the past 15 years [8,9], have been instituted only to relatively limited extents [10], and resistance has arisen. Several models of CIRBs have been suggested for multi-site studies, generally allowing local IRBs to accept, reject, or amend these CIRB reviews. But it is not clear whether such increased centralization will occur, and if so, to what degree, how, and when. These issues are significant, since the impact, outcomes, and effectiveness of CIRBs will doubtlessly affect whether they are more widely adopted.

In a recent in-depth semi-structured interview study I conducted of views and approaches toward research integrity (RI) among IRB chairs, directors, administrators, and members [11], issues concerning CIRBs repeatedly arose. The study aimed to understand how IRBs viewed RI, which these participants defined very broadly. Interviewees revealed how they defined, viewed, and addressed integrity in research, and responded to violations of RI in a wide variety of ways, related to how they saw and approached the roles and responsibilities of IRBs; interpreted and applied federal regulations; viewed and interacted with researchers, federal agencies, institutions, and industry funders; and were affected by histories of violations of RI and audits at their own and other institutions, and psychological and personality issues on their IRB [12]. They varied on whether additional guidelines and regulations would be helpful, and if so, what, where, and why; and how they related and responded to principle investigators (PIs), tried to improve these relationships, responded to tensions and complaints concerning IRBs, and interfaced with researchers in the developing world [13]. Issues frequently arose concerning how IRBs differed, and whether CIRB mechanisms might be advantageous or disadvantageous, and how, when, and why. Since the study used qualitative methods, it allowed for further detailed explorations of domains that emerged, shedding light on these issues.

A few studies have probed logistical aspects of IRBs (e.g., sociodemographics of members, and length of time that transpires before approval) [7,14,15]. One study found that CIRBs reduce time, staff effort, and costs [16], though the quality of reviews was not assessed. Yet CIRBs have encountered resistance. Among medical school IRBs, 76% have never used a CIRB, and 73% thought there was no reason to do so since their local IRB worked efficiently [17]. Surprisingly, no published studies have systematically examined how local IRBs view and experience CIRBs. Thus, this paper examines IRB chairs, staff, and members' attitudes and interactions with CIRBs - how these individuals have viewed and interfaced with these entities, and what they perceive to be the advantages and disadvantages of each of these two mechanisms for review.

## Methods

As described elsewhere [11-13], I conducted in-depth telephone interviews of 2 hours each with 46 chairs, directors, administrators, and members. I contacted the leadership of 60 IRBs around the country, representing every fourth one in the list of the top 240 institutions by NIH funding, and interviewed IRB leaders from 34 of these institutions (response rate = 55%). In some cases, I interviewed both a chair/director and an administrator from an institution (e.g., as the chair thought that the administrator might be better able to answer certain questions). From these 34 institutions, I thus interviewed a total of 39 chairs/directors and administrators. The institutions range in location, size, and public/private status. Inclusion of IRBs from a wide range of institutions allowed for illumination of the roles of different social and institutional contexts on these issues. I also asked half of these leaders (every other one on the list by amount of NIH funding) to distribute information about the study to members of their IRBs, in order to recruit 1 member of each of these IRBs to be interviewed for the study as well. Thus, in addition to the 39 chairs/directors and administrators, I interviewed 7 other members (6 regular members and 1 community member) as well.

As summarized in Table 1, these 46 individuals include 28 chairs/co-chairs; 1 IRB director; 10 administrators (including 2 directors of compliance offices); and 7 members. In all, 27 were male and 19 were female. 1 was Asian/Pacific Islander, while the remaining 43 were Caucasian. 21 came from institutions in the Northeast, 6 from the Midwest, 13 from the West, and 6 from the South. From institutions ranked 1-50, 51-100, 101-150, 151-200, 201-250 in NIH funding, the number of interviewees were 13, 13, 7, 1, and, 12, respectively.

**Table 1:** Characteristics of the Sample

	Total	% (N = 46)
**Type of IRB Staff**		

Chairs/Co-Chairs	28	60.87%

Directors	1	2.17%

Administrators	10	21.74%

Members	7	15.22%

**Gender**		

Male	27	58.70%

Female	19	41.30%

**Institution Rank**		

1-50	13	28.26%

51-100	13	28.26%

101-150	7	15.22%

151-200	1	2.17%

201-250	12	26.09%

**State vs. Private**		

State	19	41.30%

Private	27	58.70%

**Region**		

Northeast	21	45.65%

Midwest	6	13.04%

West	13	28.26%

South	6	13.04%

		

**Total # of Institutions Represented**	**34**	

The interviews focused on differences in participants' views of integrity of research, broadly defined, and in IRB responses to problems in these realms, and factors involved, and shed important light on many other, related issues as well that arose concerning IRBs' decisions, and relationships with PIs and each other, local *vs*. centralized IRBs. Interviews then explored these domains in further depth. Relevant sections of the interview guide are attached (see Appendix A), through which we sought to obtain detailed descriptions of the above issues. From a theoretical standpoint, Geertz [18] has advocated studying aspects of individuals' lives, decisions, and social situations not by imposing theoretical structures, but by trying to understand the individuals' own experiences, drawing on their own words and perspectives to obtain a "thick description."

In the methods, I adapted elements from grounded theory [19], as I have used and described in several prior studies [20-22]. This approach was thus informed by techniques of "constant comparison," in which data from different contexts are compared for similarities and differences to see if they suggest hypotheses. This technique of "constant comparison" generates new analytic categories and questions, and checks them for reasonableness. During the ongoing process of in-depth interviewing, how participants resembled or differed from each other, and how social, cultural, and medical contexts and factors contributed to these differences, are constantly considered. Grounded theory also involves both deductive and inductive thinking, building inductively from the data to an understanding of themes and patterns within the data, and deductively, drawing on frameworks from prior research and theories.

In conducting thematic content-analyses, I also triangulated methods, referring to the published literature, as described above. I drafted the questionnaire, drawing on prior research I conducted and published literature. Transcriptions and initial analyses of interviews occurred during the period in which the interviews were being conducted, enhancing validity, and these analyses helped shape subsequent interviews. The Columbia University Department of Psychiatry Institutional Review Board approved the study, and all participants gave informed consent.

Once the full set of interviews were completed, subsequent analyses were conducted in two phases, primarily by a trained research assistant (RA) and me. In phase I, the RA and I independently examined a subset of interviews to assess factors that shaped participants' experiences, identifying categories of recurrent themes and issues that were subsequently given codes. We read each interview, systematically coding blocks of text to assign "core" codes or categories (e.g., instances of IRBs interacting with CIRBs, and views of CIRBs). While reading the interviews, a topic name (or code) was inserted beside each excerpt of the interview to indicate the themes being discussed. We then worked together to reconcile these independently developed coding schemes into a single scheme. We then prepared a coding manual, defining each code and examining areas of disagreement until reaching consensus between them. New themes that did not fit into the original coding framework were discussed, and modifications were made in the manual when deemed appropriate.

In phase II of the analysis, an RA and I then independently performed content analysis of the data to identify the principal subcategories, and ranges of variation within each of the core codes. The sub-themes identified by each coder were reconciled into a single set of "secondary" codes and an elaborated set of core codes. These codes assess subcategories and other situational and social factors. Such subcategories include, e.g., perceived advantages and disadvantages of CIRBs - such as possession of local knowledge of PIs.

Codes and sub-codes were then used in analysis of all of the interviews. Two coders analyzed all interviews. Where necessary, multiple codes were used. We assessed similarities and differences between participants, examining categories that emerged, ranges of variation within categories, and variables that may be involved.

We examined areas of disagreement through closer analysis until reaching consensus. We checked regularly for consistency and accuracy in ratings by comparing earlier and later coded excerpts. To ensure that the coding schemes established for the core codes and secondary codes are both valid and reliable (i.e., consistent in meaning), they were systematically developed and well-documented. In this process, we were able to explore "cases" of problems that arise (e.g., difficult decisions IRB chairs faced), to examine the range and patterns of issues that emerge.

## Results

Several themes emerged concerning advantages and disadvantages of local *vs*. centralized IRBs. Table 2 summarizes these themes (which reflect critical codes and sub-codes used in the data analysis). In brief, all of these interviewees favored local over centralized reviews. They had all confronted challenges concerning multi-site studies, and were very aware of issues concerning CIRBs. Most had interacted with centralized, for-profit IRBs (e.g., Western IRB), and several had worked with governmental CIRBs as well. Most had attended national IRB meetings (e.g., of PRIM&R) - where these issues are often discussed - and interacted with colleagues at other institutions, and thus, had experience with CIRBs directly and/or indirectly. Overall, they offered six broad advantages of local reviews, and three limitations of CIRBs. One advantage of CIRBs and one disadvantage of local reviews also arose.

**Table 2:** Views of Local IRBs Regarding CIRB Review

Perceived Problems and Ambivalence Concerning Local IRBs
▪ Problems concerning local IRBs often recognized

▪ But general wariness of CIRBs, and support for local IRBs



Perceived Advantages of Local IRBs

▪ Claims that local IRBs reflect community values

▪ Local knowledge of subjects

◦ Of vulnerable populations

◦ Therefore, easier to judge risks and benefits

▪ Local knowledge of PIs

◦ "Track records"/reputations

▪ Protecting "our own" subjects

◦ Perceived responsibilities to protect local patients

▪ "Curbside consults" with PIs

◦ Formal and informal

◦ Can facilitate mutual trust

◦ More dialogue

◦ Appreciation of local institutional culture

▪ Desires for local autonomy, authority, and comfort

◦ Against "being told what to do"

◦ Wariness of centralized, federal bureaucracy



Perceived Problems with CIRBs

▪ Differences between CIRBs

◦ Depends on who are members of the committee

▪ For-profit CIRBs may have conflicts of interest



Advantages of CIRBs

▪ Rarely acknowledged

▪ Streamlining work

◦ Saving Time



Disadvantages of Local IRBs

▪ Discrepancies can arise due to:

◦ Institutional culture and history

◦ Personalities



Local Members as Biased in Their Views of CIRBs?



Other Possible Solutions

▪ More guidance

▪ More regional IRBs?

These interviewees' beliefs and perceptions of the advantages and disadvantages of local *vs*. centralized reviews are not necessarily objectively "accurate" and/or always justified, but are nonetheless important, shedding light on difficulties that emerge, and will no doubt continue to do so, in attempts to alter the status quo.

### Perceived Problems and Ambivalence Concerning Local IRBs

Almost all interviewees strongly defended the current system, though a few described themselves as "IRB critics," mentioning flaws in the status quo. Still, in the end, even these relative skeptics supported local approaches. As one chair said,

The whole IRB system is a mess. There are now over 5,000 IRBs in this country, and if you are a company trying to place your protocol in individual institutions, that can drive you nuts. It's terrible. **IRB4**

But ultimately, even this chair supported a local, rather than centralized or regional system, to maximize the *quality *of reviewing and protection.

However, I feel very strongly that the closer you get to the actual subjects, the better review you get, and the better you can protect the rights and safety of the subjects. **IRB4**

A few interviewees took a more balanced, and perhaps nuanced view, and felt that the status quo has both strong advantages *and *disadvantages that need to be carefully considered and weighed.

We have a very decentralized system that gives a lot of responsibility and flexibility to local IRBs, which is *a blessing and a curse*. **IRB18**

Yet in the end, these respondents, too, favored continuation of local review.

### Perceived Advantages of Local IRBs

#### Claims that local IRBs reflect community values

Several interviewees were conflicted, but ultimately felt that local IRBs were essential because these provided input concerning local community values. Frequently, interviewees claimed that local IRBs could uniquely reflect differences in local community values.

A centralized IRB system for multi-site studies *sounds *like a good idea, but the local research context and the *community attitudes *towards research still need to be checked. I'm *torn *about that, because there's tension. It would be good and efficient to have one body with all the information, providing oversight and monitoring. But somebody out there in Timbuktu could screw up, or do something inappropriate to that culture, and nobody would know. You wouldn't have a local community member saying: "This will never fly in my community." So, a centralized mechanism doesn't get you away from the problem of having to go through multiple hoops. **IRB26**

This administrator cites the importance of contrasting community attitudes, but of note, invokes the notion of "Timbuktu" - a foreign exotic locale. She uses potential cross-national variations to argue that potential domestic differences exist as well to justify local IRB review in the US.

Occasionally, interviewees offered examples of what they felt were differences between communities. For instance, one chair in a rural area saw a contrast between rural and urban areas, each having varying perceptions of research. He thought that patients in his community were more likely to decline participation.

In my own research, it is very difficult to recruit people for anything invasive. In the city where I trained, we did the same level of invasive studies, but people just kind of understood this research: I'm volunteering. *Here*, you have to explain yourself more: this is what research means, this is the importance of doing it. Not with everybody. But with the occasional study participant, it just takes a little more effort to give them the confidence that it's OK. A lot of it has to do with it being a rural community - less of a big city. Here, everybody knows each other. But research just isn't understood as in a big urban center, due to exposure in everyday life. Here, the newspaper has articles about farming, and on a far distant page, about science. **IRB27**

Yet, the differences he suggests appear to be due to awareness and education, not necessarily contrasts in underlying community *values per se*. Rural and urban regions may also range in logistical factors. He continued,

There's sort of an identity here of rugged *individualism*, but I don't think that's what gets in the way. It's just lack of awareness, exposure, and understanding of the importance of research. Part of it though is that some people would have to drive three hours to get here, and that's just not feasible. **IRB27**

Thus, perceived variations may arise not from differences in local moral values, but from other geographic factors. Indeed, the same proportion of the population may be interested in research in both locales, but rural areas simply have fewer patients overall. Hence, more populous regions have higher absolute numbers of interested participants. Yet no interviewees provided clear instances of differences in local community values.

#### Local knowledge of subjects

In supporting local, as opposed to centralized, boards, almost all interviewees cited advantages of "local knowledge" - e.g., knowing about local subjects. Indeed, only a local IRB may know certain unstated details about a protocol, such as the fact that a particular institution primarily serves a disadvantaged population.

In one study of parenting skills for moms, the rationale was to show how young moms, living alone without the benefit of several generations to help them, didn't know anything about comfort care - lullaby singing, story-telling, rocking chairs with fussy babies. But the hospital they picked was a private hospital that only accepts private pay patients. The patients who could benefit most from the study were indigent patients in clinics. We asked: are those individuals really going to be at this private hospital? One co-investigator saw patients only at that hospital, and had quite a bit of influence. We urged them - we cannot insist that a study be conducted at a specific hospital. But if the objectives are for a particular population who can't take advantage of the study, I think, it's within the IRB purview to say, would you be willing to offer this study to people who are not patients there - advertise it through clinics or hospitals where these other patients would be able to access it? On the surface, the protocol met almost all of the requirements. Their objective was well stated, but that's where the local oversight comes in - if you didn't know that that hospital only accepted private pay patients. **IRB13**

Only a local IRB might know that a particular hospital serves a population that is vulnerable in other ways as well, and that *extra *protections might thus be needed. As another chair explained,

If I am going to recruit all my patients from a certain psychiatric center, a colleague here in town would know *exactly *what I'm talking about, and who those patients are - and their demographics. That kind of *local knowledge*. If you are accused of a crime, you want to be judged by *a jury of your peers*, not by somebody who has the same demographic from halfway across the country. **IRB12**

This chair draws analogies to juries that presumably consist of one's peers. He suggests that "peers" are based not on demographics alone, but on other characteristics - shared knowledge of a community. This chair sees importance in knowing details about a population - having a sense of familiarity that is concrete, not abstract.

The farther away you get from the actual group of subjects, *the harder it is for a committee to judge the risks and benefits*. So in our IRB, we are thinking about whether it's ethical and reasonable to do a study of new asthma treatments for young black children in our city. This is not abstract. We all know exactly who's involved, what *the lives *of those individuals are like, what the protocol could offer them. An IRB in another state could not make as informed a decision. **IRB12**

Ideally, centralized or regional IRBs should be able to request and assess such details, but his comment highlights challenges CIRBs may face.

#### Local Knowledge of PIs

Local knowledge can also extend to IRBs knowing local PIs' individual characters, personalities, and past experiences.

The most effective IRBs are those in an institution where the reviewers are familiar with the work and integrity of the researcher. That is the most important element, other than the actual research proposal itself. What's this researcher's *track record*? Has he been shot down once or twice? Regional IRBs might get reports of that info, but don't have the personal understanding, *feel*, and *flavor *that's needed for a heightened level of review. Regional IRBs may be administratively effective and sound, but focus on cooperative group research, clinical trial groups, cancer groups. They are good for a subset of clinical trials. **IRB9**

This interviewee suggests a higher standard here - a "heightened level of review" - that only a local IRB can provide. Yet questions arise of what this level consists of; how "heightened" it is; when it is needed and/or feasible, and to what degrees; and in what ways knowledge of the PI does or should influence reviews.

In reviews, notions of "feel" and "flavor" thus arise, suggesting subjective, almost aesthetic qualities involved. Such local knowledge about PIs can include subjective impressions and suspicions concerning their integrity and views of, and respect for the IRB. For instance, local PIs may try to use their power and influence to affect IRB decisions, which can then make the IRB wary about future interactions. An IRB may feel that these efforts shape deliberations and indicate deep disrespect.

When a PI tried to exercise his power, as a vice-dean, and convince the IRB to review a study favorably, his future submissions were questioned. It's an *integrity *issue: he's exercising a position of influence, which contributes to the appearance of *coercion*. That's when the research Compliance Office comes in. That happens rarely - not in three years. It's usually low risk research. The PI may use the "*no harm, no foul*" principle: "I did all this research without IRB review. I now know I need IRB approval. But no one got hurt. What's the big deal? *Give me retroactive approval*." Obviously, we can't. But it doesn't stop there. One person came back and said, "You're going to screw me up, ruin my publication, tarnish my reputation." *Mea culpa *is one thing. We approve use of the data, but don't give retro-approval. We are sympathetic, but can't bend to that type of coercion. An IRB decision cannot be overturned by anyone in the institution. That's the independence given by the regulations. **IRB9**

The IRB may then examine that researcher's subsequent studies more closely. This IRB director continued,

We look at that PI's future research a little more cautiously. Because that PI is willing to try and convince his peers to make an exception for him because of who he or she is. **IRB9**

Personal knowledge of a local PI can thus potentially sway assessments of a protocol, yet prejudice may exist either positively or negatively. Questions then surface as to how important that past experience is or should be - whether such added future vigilance might ever go too far, and whether each protocol should instead be reviewed on its own merits alone (as with blinded peer-reviews of scientific journal articles), unaffected by whom the researcher happens to be.

Moreover, in reviewing a protocol, personally knowing the PI can potentially not only help, but create tensions, since the IRB may then feel explicit or implicit, and direct or indirect pressure to approve his or her study.

#### Protecting "our own" subjects

Many interviewees felt a particular bond and responsibility to protect the patients at their own institution. In part, as clinicians, many interviewees felt obligations toward their patients.

With the local approach, we absolutely feel committed to the subjects in this area. A lot of them are *our patients*. They are from the community that we are committed to serving. That commitment couldn't be higher. I would worry that if the IRB became more regionalized, de-personalized and, particularly, for-profit, that would go away at little bit. I am not sure. **IRB40**

Thus, for IRBs, a sense of professional, social, and geographic closeness at times appeared to heighten a sense of moral obligation. As a result, interviewees expressed added wariness of CIRBs.

As suggested earlier, IRBs often feared centralized bodies as "anonymous bureaucrats in Washington." This attitude reflects in part a sense of bonds, and being part of a local community, but also stemmed from a general wariness of federal government involvement, with its innate anonymity.

Centralized IRBs may be a solution, but we really want to know what's going on on *our campus *- what's being said, conveyed to *our community*, and participants. It would be hard for people to say, "Oh, this was approved by some anonymous body, so it should be OK." **IRB27**

#### "Curbside consults" with PIs

Though federal regulations established IRBs as local, presumably to reflect local laws and standards, unintended benefits emerge - that PIs can interface with the IRB both formally and informally, facilitating interactions. Knowing the local "gatekeeper" (i.e., the IRB) has advantages.

The concept of local IRBs was to get local cultural input into the review of studies in a particular community. I suspect that that's less of an issue in very white and affluent areas like ours. It might be a very big issue in Alabama. I think Tuskegee could have been very different. So, the basic *philosophical *premise of local IRBs is very good. I like the decentralization, not because I like repeating all the work, but because I'm not sure I would *trust *a central IRB somewhere in Washington. I don't think it was the intent, but the *practical *outcome of local IRBs is that *PIs bounce stuff off me all the time*. The concept of an IRB helping to inform the design of research as well as being a *gatekeeper *is good, though I'm not sure that was ever intended. **IRB14**

IRBs can thus interact with PIs informally and in person, rather than through the constrictions of formal memos back and forth. Such informal verbal communication can allow PIs to present possible approaches (e.g., "what if I did X?"), while inherent constrictions of formal memos might impede such interactions. Informal conversations can thus help shape studies early on, clarifying what research approaches may be ethically problematic before they are more elaborately pursued. This interviewee also mentions the underlying desire for *trust *- of CIRBs and PIs - which is important, but can be fragile. Informal local feedback has the advantage, too, of being readily available.

Researchers say, "What do you think of this?" I say, "I can tell you: 'We're going to make you do this,' 'say X.' 'No, you can't give the pharmacist a finder's fee to identify how many patients are getting this drug.'" Or, we'll get an informed consent, and say, "Our IRB likes to see the wording a certain way. Email me, and we'll send it back to you." It is good to have the *local cooperative mechanism*. **IRB14**

Trust and a sense of mutual commitment can thus be increased.

Curbside consults can allow PIs, too, to explore the possibility of changing protocols already approved - e.g., making exceptions to include patients who do not exactly fit the pre-established criteria or timeline. These changes might not alter the direct risks or benefits to the subject, but the indirect social benefits of the research, about which local IRBs can thus readily inform PIs.

The most frequent *curbside *thing I have to deflect, and say OK, we need paperwork for this, is: "This patient fits the study really well, except for this *one really little glitch*. Can I put him in the study anyway? The protocol says within four weeks. It's been four weeks and three days." That's when I push back. With investigator-initiated studies, researchers then say, "It doesn't really make any difference, but I had to write something in the protocol." I answer: "If that rule was important enough to write down, why is it OK to break it? If it's OK to break it, maybe you should rewrite the rule to reflect *reality*." I do that a lot - not so much trying to protect the subject from risk, but *protecting the science from damage*. If you make all these exemptions, you are going to dilute the science...If you start to waffle, and get uninterruptible results, it puts the science at risk. Usually, it's not serious, but a couple times I've said, "This is the fifth time you've asked me about this. Rewrite the protocol, rather than making all these exemptions. If this is an important rule, you can't break it." They then have to convince me that it's OK. Because otherwise, thinking ahead, the paper's method section says "patients will have had a CT scan no more than four weeks ago." And I'll say, "That's not true." This is small stuff, but it's of a piece of real falsification of data. **IRB14**

Though the *content *of the disagreement here does not concern differences in local community values, local IRBs can potentially facilitate the *process *of interactions, allowing far more informal "give and take." These closer and more frequent interactions can also enhance PIs' education, and potentially diminish the likelihood of PIs making potentially problematic changes without notifying the IRB.

Local chairs' interactions with colleagues can enable the former, too, to hear about researcher's difficulties and struggles, and may enhance IRBs' understandings of the strains researchers confront.

I hobnob with colleagues. I hear the woes of our local cancer investigator over audits in big cooperative studies. **IRB14**

Improved mutual understanding between IRBs and PIs can help mollify ongoing tensions that can exist.

At institutions that focus on just one medical specialty, disease, or organ - whether cancer or diabetes - IRBs will also understand this specific area of medicine, which can be helpful. As one interviewee from such a specialty institution said:

A lot of researchers here would be upset if we did away with our IRB. Here, people understand this specific area of research - the terms and concepts. **IRB8**

Still, regulations mandate that all IRBs have appropriate specialization for reviewing all protocols.

#### Desires for local autonomy, authority, and comfort

IRBs may also want to review a protocol on their own because they need to undergo their own cognitive and emotional processes to become familiar and comfortable with the issues involved.

Each IRB has *its own learning curve*, and comes to its own sense about an issue. I could call up the IRB administrator and say, "We've been through this. This is how we see it. This is how it is." They would say, "Thank you very much. We're going to go figure it out for ourselves." Like anybody, *we don't like other people telling us how to run our own shop *- although it should count for something that another IRB has approved it. I don't know how you can streamline that. It is inevitable. **IRB26**

IRBs' decisions can thus involve complex cognitive and emotional processes, and IRBs may not like to be told how to proceed. However, this statement suggests, too, that local control and potentially liability, not local community values, may at least in part underlie IRB preferences for local review.

### Perceived Problems with CIRBs

#### Differences between CIRBs

Several interviewees had had experience with more than one CIRB, particularly for cancer trials with adults or children, and perceived critical variations. Problems arose with these entities, due not to the underlying concept of such centralization, but to the specific realities of *who *exactly is on each board, what training they have had, and how "reasonable" they were. Chairs and members of CIRBs, like those of local IRBs, could have their *own *personalities and idiosyncrasies that can shape their approaches to studies.

Two-tiered models exist - whereby local institutions can accept, reject, or modify a CIRB review - but can still pose challenges.

I don't want to lose individual input at the level where the study takes place. The state IRB, established by NCI with adults, represents one of the more logical approaches to review, taking relatively little time, and still allowing local input. We still have an option of altering, overriding, or rejecting the central CIRB action. **IRB4**

Yet individual CIRBs may themselves differ significantly. A more centralized IRB could even be *stricter *than a particular local one. One city-wide IRB was more obsessive than one institution's own board.

In our city, research involving people covered by Medicaid and Medicare has to go through a city IRB, which puts more control on you. It requires more and more time and paperwork, and can get crazy, particularly for behavioral research. They often set an even *tighter *set of rules, and just keep coming back with more questions in whatever you do. The city IRB is primarily laypeople, and they are very, very thorough. Some of these members have four pages of handwritten notes on a very small study. It's too much. **IRB25**

Similarly, a few chairs feel that the centralized pediatric cancer IRB is not as good as the adult one.

A model is only as good as the quality of the reviewer. I have absolutely rejected, refuse to become a part of a similar program with Children's Oncology Group because the quality just isn't there. **IRB4**

This chair felt that the pediatric CIRB ended up with overly complicated consent forms.

Children's Oncology Group's IRB has a sort of *dictator *syndrome. No one gives them feedback, while the *adult *CIRB listens to feedback and is not like that. **IRB4**

Each IRB, even if centralized, may vary, and have particular approaches and notions concerning informing the participants. This chair continued:

In pediatrics, the quality of the consent forms - the way they present information - pales in comparison to the adult CIRB's. The Children's Group forms are not *user-friendly enough*, and do not convey information as it could be. Perhaps the pediatric group has bent over too far backwards, offering complex charts, with medical jargon of potential drug side effects. Some of the consent forms just leave my head spinning. They present *facts*, not *information*. **IRB4**

This distinction of facts *vs*. information highlights the different functions of each. Information is meant to "inform," to create a sense of understanding in the recipient's mind, while "facts" implies nothing about the recipient whatsoever. Yet to decide how to "inform" *vs*. merely "present facts," and what is "user-friendly enough" can be hard, and in the end, somewhat subjective.

Several other chairs also preferred the adult, rather than the pediatric, CIRB, and the fact that IRBs can choose to accept or alter the adult board's reviews, prompting support of at least some centralization.

CIRB pass-throughs, mostly from the NCI's cooperative group studies, have helped streamline our work. The discussion notes are high quality, and we can choose to accept their reviews. That would be a good way of reviewing studies regularly. **IRB14**

The differences though between the adult and pediatric CIRBs may result in part from historical reasons. It was noted that the adult CIRB took time and sought input, while the pediatric group appeared pressured to quickly do the same.

Four years before the adult CIRB launched, they asked for *input*. They had draft after draft of how to go about doing what they wanted to do. They did it very cautiously, with relatively few members, to test the waters, and it turned out very well. I think the Children's Oncology Group was simply pressured to follow suit, but did so without having the requisite background and preparation. **IRB4**

Perceptions of the pros and cons of CIRBs depend in large part, too, on the specifics of how much local IRBs are subsequently permitted to modify or reject reviews.

We've worked very well with the adult CIRB because it leaves the local IRBs a lot of room to make changes. We're very happy with that. Other large-scale regional IRBs have not seemed all that great. Limited numbers of people have oversight, and are not involved with the institution. They don't know the *institutional*, let alone the local, culture. The adult CIRB differs from the others. It's national, looking at huge cancer studies all over the country; so they develop a *standard *of what they expect, and what's in the consent. Local IRBs can then modify it, which they've allowed. It's probably very effective. But the larger regional IRBs are more focused on the *specifics *of the study, and tend to do more with the study itself. **IRB25**

Hence, the *details *of how more centralized models work are important. The debate thus needs to evolve beyond simply central *vs*. local, to examine to what degree, and how exactly different models might operate. This interviewee feels that knowledge of local institutional culture is important as well (though not the intent of the regulations).

Multi-site studies may also vary in structure, such that a primary site may simply take the lead, and other IRBs might thereby cede to it - though not always. An institution could potentially agree in advance to rely on another institution's IRB, but then disagree with a particular review. One IRB agreed to make an exception, and accept an outside review because this IRB was an "add-on," and did not want to be "the tail wagging the dog."

We accepted their consent form and protocol, and told our investigator, "You don't have to redo it." It was excellent. But that was an *exception *to the rule...Really the *only *one, because we were at the point, otherwise, of being the tail wagging the dog. The other medical center was taking the lead, and doing most of the research. Our researchers just sort of tagged along. We said, "Oh well, we'll make an exception in this case." **IRB8**

Local IRBs may simply *not want *to cede much, if any, control to a more central institution. Ceding responsibility may be hard legally, ethically, and potentially emotionally, raising fears and anxieties about losing control.

Interviewees ranged, too, in their views of for-profit CIRBs (e.g., Western IRB). While some individuals here relied on and respected such entities, other interviewees questioned how these bodies could be sufficiently aware of local communities and values. Concerns arose, too, that for-profit entities might have conflicts of interest, since they are paid to review and approve studies.

### Advantages of CIRBs

As suggested above, interviewees explicitly acknowledged advantages of CIRBs only rarely, but implicitly potential advantages as well. Specifically, CIRBs with a local option to accept, reject, or modify reviews could "streamline" work, and save time.

### Disadvantages of Local IRBs

Interviewees described local IRBs as varying in their "colors" and "flavors," and in how "nitpicky" *vs*. "user-friendly" and "pro-research" they were, based not on local community values, but on characteristics of the chair and/or vocal members (e.g., whether they were themselves researchers), and local institutional cultures and histories. Several IRBs appeared more cautious or "obsessive" because of experiences and/or fears of federal audits or "shut-downs" of research at their institution in the past 10 to 15 years (causing "The Reign of Terror"). Yet interviewees generally accepted such differences between local boards as inherent aspects of the system.

### Local IRB Members as Biased in Their Views of CIRBs?

At times, in part due to the psychological and legal issues mentioned earlier, IRBs appeared *biased *in favor of local control. In articulating the benefits of local review, interviewees often seemed to have little, if any, sense of the status quo's potential costs to investigators. The chair who stated earlier that it was easier to judge the risks and benefits that are closer (*vs*. "farther away") added:

I like the current model. I'm not sure why. I've never been on regional IRBs. **IRB12**

He continued that in an inter-institutional research group, in which he is engaged, he doesn't find many variations between IRBs, except merely over wording.

As an investigator, I'm involved in a consortium, and don't see a lot of difference between what's acceptable at one institution *vs*. another. There are usually arguments over wording here and there, but not the basic risk *vs*. benefit analyses of a protocol. **IRB12**

Though one might expect all researchers in multi-site studies to favor CIRBs, he does not, perhaps because he has long been an IRB member. Yet his statement undermines his earlier notion that local community values play important roles here, and conflicts with the data, mentioned earlier, that discrepancies often involve more than wording alone. He and others also do not appear to consider or weigh possible logistical and other costs to PIs involved.

### Other Possible Solutions

#### More guidance

Several interviewees were wary of CIRBs, and felt that more guidance, not CIRBs, can help reduce ambiguities in current regulations, and solve ongoing problems and tensions in the present system.

Investigators think a central IRB will make things go faster. But it doesn't really solve problems. The 14 or so pages of regulations reflect the philosophy of the federal government in the 1700s or 1800s: it's all about *local control*. Local control is good on a school board. It isn't very good when researchers across the country are trying to conduct the same study, but need to meet different standards. That takes a lot of time, and introduces a lot of noise, and variation in the consent, the review process, and the regulatory oversight. The federal government or OHRP needs to step in, and give some *guidance*. They don't have to say, "It's gotta be *this *way," but they have to provide *more guidance *than there is now. The regulations basically say: it's up to you to interpret things. But what is slight increase over minimal risk in pediatric studies? On a whole host of issues, we have absolutely no guidance, which contributes to very heterogeneous reactions of IRBs handling exactly the same studies. **IRB5**

#### More regional IRBs?

In the debates over whether to establish more centralized or regionalized IRBs, several interviewees suggested that one alternative would be simply to have more regional IRBs, gathering more local information. For instance, the chair who saw a current "city-wide" IRB (which reviews only certain, but not all protocols) as "too strict" nonetheless perceived potential advantages in wider use of such municipal-wide entities - though not this *particular *one.

We have so many institutions here, that it would be interesting to have a city-wide IRB. **IRB25**

## Conclusions

Interviewees tended to have strong views concerning CIRBs, and highlighted advantages and disadvantages of both local and centralized processes. Overall, however, these local IRB chairs, administrators, and members overwhelmingly supported local over centralized IRBs. Specifically, these interviewees argued that local IRBs can better provide local knowledge of subjects and of PIs, and "curbside consults" with PIs that can facilitate mutual trust, potentially decreasing non-compliance. Local IRBs may also appreciate local institutional culture. While a few interviewees acknowledged that the current system can impede multi-site studies, and that CIRBs could potentially reduce duplication of efforts and streamline work, in the end all interviewees supported the continuation of local IRBs in some form.

Importantly, these interviewees also suggested that the quality of CIRBs can range considerably. Many of these IRBs had had experience with the centralized reviews of the NCI for adult oncology trials, and of the Pediatric Oncology Group, and of for-profit IRBs, and felt that the nature and outcome of centralized IRBs can vary, depending on who happens to be members of the committee. Of note, both local and centralized IRBs appear to have unintended consequences, both good and bad, that have received little attention, and need to be more fully taken into account.

These interviewees felt that the local knowledge of subjects and PIs, long-standing informal interactions with these PIs, and consequent trust and IRB "comfort" are important. PIs may interact more fully and informally, and hence in many ways effectively, with local than centralized IRBs. In general, interactions can depend on both the quantity and quality of information exchanged. Communication early in a process can lead to avoidance of later difficulties (e.g., expending efforts on what turn out to be unfruitful approaches in addressing research ethics issues).

IRBs here believe that accessibility to PIs may enhance *trust*. Local IRBs can potentially play vital roles in providing "curbside consults" that may establish and maintain mutual trust that may, in turn, help ensure the protection of human subjects. Hence, *informal *interactions in an institution, established over time, can be critical. However, future research is needed to explore how PIs themselves view these issues - whether they agree, and what factors they feel increase or decrease their trust of IRBs.

Though usually seen in debates as fixed, objective, bureaucratic entities, IRBs emerge here as engaged in complex, dynamic social systems, shaped by particularized, individual relationships with PIs, institutions, and communities.

These social bonds include not only colleagues and PIs, but patient populations as well. IRB chairs and members, many of whom are themselves physicians, felt a sense of commitment to "their patients" - i.e., those of their institution - and felt that entrusting these patients to a distant, "anonymous" CIRB represented an abdication of this responsibility. This sense of commitment highlights how belonging to a community can generate deeply personal social, as well as moral, bonds that can potentially enhance protection of subjects. Belief in this sense of local bonds may fuel local resistance to CIRBs. But presumably, an ethical obligation to safeguard research subjects as much as possible should exist on CIRBs as well - i.e., be the same for participants elsewhere, too. Researchers should presumably not protect subjects at their home institution more than participants at another medical center.

The close knowledge of PIs and patients that local IRBs offer does not appear to have been the intent of the regulations. Rather, such IRBs were established to reflect local community laws and values. But between IRBs, discrepancies that arise may result instead from differences in local knowledge, or institutional history and culture, and personalities and idiosyncrasies of particular IRB chairs and/or vocal members, or may be far more complex and seem relatively random - i.e., not systematically resulting from clear, objectifiable factors. Further research is needed as well to probe more fully these potential underlying causes of discrepancies.

High levels of communication and trust between IRBs and PIs, and IRB knowledge of details about subject populations, and commitment to these individuals, are nonetheless valuable. But difficult tradeoffs then ensue. Local IRBs may enhance reviews (e.g., providing local knowledge of PIs) and/or generate costly and unnecessary discrepancies. However, it is not clear how to balance these advantages (which were not the intent of the regulations) against the disadvantages of local IRB reviews (e.g., inter-IRB discrepancies). Whether these advantages of local IRB review offset the resultant problems, and whether a system can be implemented that yields these benefits, while avoiding the disadvantages of local review, is not evident. Needs emerge to ensure that details of local knowledge are sufficiently incorporated into reviews, but how to do so such that reviews do not also become duplicative is unclear.

Within the US, it is unknown, too, how often community values significantly differ between IRBs in ways that do or should shape specific IRB decisions. No systematic data have ever been published concerning this question. The fact that institutional and personality issues can play roles is not surprising, given the complex dynamic social processes involved. But while interviewees, in arguing for local IRB review, frequently aver that variations exist in community values, the examples that the men and women provide here appear instead often to reflect differences in local knowledge, and potentially other factors. Further research is thus vital on other, larger samples concerning how often, in what ways, and to what degree inter-IRB discrepancies result from differences in local values *vs*. local knowledge and other causes.

These interviewees' reasons for favoring local IRBs reflect attitudes (e.g., local knowledge of PIs and study populations), rather than objective evidence of improved human subject protection. Yet some of these attitudinal factors may influence protection of human subjects (the goal of IRB review) not directly, but indirectly. Systematic studies demonstrating the degrees to which IRBs in fact reduce concrete harms to subjects are still lacking. Granted, such evidence may be difficult to collect since such injuries related to deficiencies in research ethics may be relatively rare and hard to measure. Subjects may be hurt due to serious, unanticipated adverse events in a study protocol (e.g., previously unknown side effects of an experimental drug), but these may not be due to deficiencies in research ethics *per se*.

Nonetheless, enhanced local knowledge of subject populations and PIs (e.g., awareness that certain populations are vulnerable, and that certain PIs have been relatively more "cavalier" in the past) is important as it can facilitate human subject protection by raising caution in reviewing certain protocols. Such increased cognizance may heighten human protection indirectly - even if not directly and measurably - by decreasing the likelihood of threats to research ethics occurring.

Relatedly, at first glance, two findings here may appear contradictory: on the one hand, interviewees support local IRBs, not because such local entities reflect community values, but because such local IRBs can facilitate interactions with PIs and provide local knowledge of PIs and subjects. Yet on the other hand, these interviewees are nonetheless wary that CIRBs may insufficiently attend to local values. In the end, however, these two sets of beliefs are not mutually exclusive. Rather, the existence of significant differences in local community values may be somewhat illusory, more than empirically-based. Despite this absence of empirical evidence, interviewees still support this rationale, because it is stipulated in 45-CFR-46, and appears to justify their existence, efforts, and expense.

These interviews also underscore several potential disadvantages of CIRBs. While some critics of IRBs may see these centralized entities as panaceas, certain problems appear likely to continue. CIRBs may be as subjective as local IRBs - i.e., shaped by the particular views, biases, or predilections of whoever happens to be the chairs and membership (e.g., how "nit-picky" *vs*. "user-friendly" and pro-research these individuals are).

These interviewees express, too, deep wariness and suspicion of CIRBs, and of "anonymous government bureaucracies," reflecting the same apprehension that underlies broader political caution of federal government involvement and notions of "states' rights." Yet as a result, local IRBs may resist efforts at centralization, potentially impeding the spread, efficiencies, and acceptance of such efforts. Given tensions due to interviewees' concerns - whether valid or not - that CIRBs will insufficiently incorporate local knowledge, other two-tiered models may be advantageous. Such mechanisms might still include some local review (e.g., having local reviewers contribute to CIRB discussions). But how such mechanisms would operate - e.g., in multi-site studies being reviewed by a CIRB - and how much such "dual reviews" would decrease expenses and eliminate discrepancies, are unclear.

Granted, as members of *local *IRBs, these interviewees may, in certain ways, be biased in their assessments. The IRB chairs and administrators here receive support - even if it is only part of their salary - for their work in the status quo, which they thus may be invested in maintaining. Future research on IRB members' views on these issues should take this potential limitation into account, and explore it more fully.

These data have additional implications for policy as well. Current debates about local *vs*. central reviews do not appear to reflect the complexities here, but should. These debates need to shift from focusing simply on a binary decision of local *vs*. centralized (or regionalized) IRBs, to explore specifically how different models do and could function; what the relative advantages and disadvantages of each are, and how to balance these; what obstacles might arise in attempting to institute any change; and whether and how these barriers ought to be overcome. Policy-makers and others thus need to carefully consider the difficult tradeoffs that emerge here (e.g., when, if ever, the benefits of local IRB knowledge offset the delays and obstacles of multiple local IRB reviews). The debate has not generally addressed these dilemmas, but needs to do so to advance future discussions.

Policy questions surface as well concerning whether, how much, and in what ways to arrange for central and regional IRBs to interact with PIs closely, frequently, and effectively. At local IRBs, these interactions may be based on long-standing relationships and mutual trust, allowing for informality that can facilitate communication. Whether centralized IRBs can also establish such relationships is critical. To do so at a federal level may be impossible, since these interactions may then diminish "informality". The responses of a federal bureaucrat would presumably be "official" in a way that local "curbside consults" are not. But potentially, CIRB reviews can incorporate additional local knowledge in varying ways and to varying degrees, and hence, review options need not be simply dichotomous - local *vs*. central, all or none. Rather, enhanced local input and reviews could be made available to CIRBs.

Many of the characteristics of CIRBs explored here apply, too, to regional IRBs, which appear more common in other countries, and seem a logical middle-ground - permitting "local knowledge" and trust, while still streamlining the review process. But the current data suggest possible limitations of such a regional system as well. Vast differences can arise in regional IRBs, depending on exactly *how *regional these entities are (e.g., whether they cover a city, state, or large part of an entire country). These interviews suggest needs for additional research and guidance on, and possible further establishment of, such regional IRBs. The specific degrees to which such regional organizations indeed provide the advantages and avoid the disadvantages of both local and central IRBs require close investigation.

Questions emerge, too, as to which studies should be reviewed through central, regional, or local boards. While some critics recommend CIRBs for multi-site studies (where the costs of multiple, conflicting IRB reviews appear highest), single-site studies might then be left wholly to local review, and prey to local biases, and potential impediments. Subjects in single-site and multi-site studies may then receive different kinds or degrees of protection. Hence, it may make sense to consider CIRBs for single-site studies as well, though doing so may be harder to justify. These issues require further examination and discussion as well.

Given interviewees' resistance to CIRBs as *structural *solutions in efforts to improve IRBs, altering local IRB attitudes may be more achievable, and hence in need of further consideration. For instance, helping IRB members to become more cognizant of their own potential biases and assumptions that are not evidence-based might be beneficial.

These data have several additional implications for future research as well. Several "myths" about both local and central IRBs - concerning their respective advantages and disadvantages - may exist. As policy-makers and others contemplate increased CIRBs, it is critical to have as full an understanding of the potential pros and cons of these entities as possible. Studies are needed to explore more fully, among larger samples, how IRB chairs, members, and staff view and interact with local and centralized boards; what the roles and effects of *informal *communication are; how frequently and in what ways local IRBs in fact alter CIRB recommendations; how to develop and assess such models of CIRBs to enhance trust and overcome wariness; and how to guide the development of any such policy initiatives.

Further research is crucial, too, to assess how often IRBs interpret and apply regulations differently in ways that reflect psychological and institutional issues *vs*. differences in community values, and whether and how educational or other interventions can make IRB interpretations more uniform and less discrepant due to personalities and local institutional histories. Further data on actual IRB practices *vs*. assumptions can help guide these debates.

This study has several potential limitations. These data are based on in-depth interviews with individual IRB members and chairs, and did not include direct observation of IRBs making decisions, or investigation of IRB written records. Future research can also observe IRBs and examine such records. However, such additional data may be difficult to obtain since, anecdotally, IRBs have at times required that researchers obtain consent from all IRB members, as well as from the PIs and funders of protocols.

These interviews probed respondents' views at present and in the recent past, but not prospectively over time to assess whether they changed their views, and if so, why. Future research can explore these issues as well.

It is possible that response bias may have occurred here as well - i.e., that individuals who agreed to participate may differ systematically from those who did not. Those who responded may be more or less favorably inclined toward the status quo. However, there is no evidence that such differences exist either way. Further research can, however, also examine these possibilities among additional, larger samples of IRB chairs and members.

This study also did not assess views of CIRB chairs or members, but future research can examine their perspectives as well. In addition, among interviewees here, experiences with CIRBs varied in amount and type - e.g., from for-profit IRBs to governmental, from extensive to minimal interactions (i.e., from dealing with one to more than one CIRB, concerning one to more than one protocol), and from harmonious to problematic and conflictual. A few interviewees did not have direct experience, but knew of other IRB members or chairs at their own or at other institutions who had had such interactions.

Nonetheless, the attitudes of all of these interviewees are vital, since these may reflect prevalent views among local IRBs more generally, and highlight wider concerns, fears, and misconceptions. These perspectives are all critical, and need to be addressed, as efforts to centralize IRBs will no doubt continue.

In sum, these data highlight the intricate, complex tradeoffs and uncertainties that may be involved in assessing the advantages, disadvantages, and varieties of CIRBs, which should be considered in ongoing discussions concerning these and other approaches to improving human research subject protection. While some critics herald CIRBs as a virtual cure-all to current problems with IRBs, interviewees here underscore the importance of advancing the current debate from whether local *or *centralized IRBs should be instituted, to more nuanced analyses of *how *these and other models may best be used. Careful ongoing investigation and discussion of these issues are vital.

## Competing interests

The author declares that he has no competing interests.

## Authors' contributions

RK designed and conducted the study, and wrote and approved the final manuscript.

## Appendix

### Sample Questions from Semi-Structured Interview

*Note: Additional follow-up questions were asked, as appropriate, with each participant*.

▪ How do you define research integrity (RI)? How has your IRB approached RI issues? What have been the most difficult cases concerning RI that your IRB has faced? How did your IRB respond?

▪ Do you think IRBs differ in their views or approaches toward RI, and if so, when, how, and why? Do you think IRBs apply standards regarding RI differently, and if so, when, how, and why?

▪ What factors do you think affect how IRBs make decisions about RI and other areas?

▪ What do you think makes an IRB work well or not in monitoring and responding to RI?

▪ Does your IRB encounter tensions with PIs about RI or other related issues? If so, how, when, and why?

▪ Is your IRB more cautious about some researchers than others? Why?

▪ What kinds of conflicts, if any, has your IRB faced with your institution?

▪ Do you think a centralized IRB rather than local IRBs would have advantages concerning RI and other areas? If so, what, how, and why?

▪ Should other regulations or guidelines concerning IRB reviews of RI or other areas be developed, and if so, what?

▪ What do you think could be done to improve interactions with PIs concerning RI and related issues?

▪ Do you have any other thoughts about these issues?

## Pre-publication history

The pre-publication history for this paper can be accessed here:

http://www.biomedcentral.com/1472-6939/12/13/prepub
